# Utility of plasma Neurofilament light as a diagnostic and prognostic biomarker of the postural instability gait disorder motor subtype in early Parkinson’s disease

**DOI:** 10.1186/s13024-020-00385-5

**Published:** 2020-06-05

**Authors:** Adeline Su Lyn Ng, Yi Jayne Tan, Alisa Cui Wen Yong, Seyed Ehsan Saffari, Zhonghao Lu, Ebonne Yulin Ng, Samuel Yong Ern Ng, Nicole Shuang Yu Chia, Xinyi Choi, Dede Heng, Shermyn Neo, Zheyu Xu, Nicole Chwee Har Keong, Kay Yaw Tay, Wing Lok Au, Louis Chew Seng Tan, Eng-King Tan

**Affiliations:** 1 Department of Neurology, National Neuroscience Institute, Tan Tock Seng Hospital, 11 Jalan Tan Tock Seng, Bukit Merah, 308433 Singapore; 2 Neuroscience and Behavioural Disorders Program, Duke-NUS Medical School, 8 College Road, Bukit Merah, 169857 Singapore; 3 Center for Quantitative Medicine, Duke-NUS Medical School, Bukit Merah, Singapore; 4 Department of Neurology, National Neuroscience Institute, Singapore General Hospital, 20 College Road, Bukit Merah, 169856 Singapore; 5 Department of Neurosurgery, National Neuroscience Institute, Tan Tock Seng Hospital, 11 Jalan Tan Tock Seng, Bukit Merah, 308433 Singapore

**Keywords:** Parkinson’s disease, Motor subtype, PIGD, Biomarkers, Neurofilament light chain, Cognition

## Abstract

**Background:**

The main motor subtypes of Parkinson’s disease (PD) include tremor-dominant (TD) and postural instability gait disorder (PIGD), with varying disease course that warrant the development of biomarkers capable of predicting progression according to motor subtype. The PIGD subtype is associated with a poorer prognosis, hence identification of a biomarker associated with PIGD is clinically relevant. Neurofilament light (NfL) chain is a potential biomarker of disease severity in neurological disorders including PD. However, no study has investigated NfL and PD motor subtypes. Here, we aimed to investigate the diagnostic and prognostic utility of plasma NfL for PD motor subtypes in early Parkinson’s disease. Given the higher risk for cognitive and motor decline in PIGD, we hypothesized that plasma NfL is a potential biomarker for PIGD.

**Methods:**

Plasma NfL was measured in 199 participants (149 PD and 50 healthy controls, HC) using an ultrasensitive single molecule array. Patients were classified into TD or PIGD based on MDS-UPDRS components. After 2 years, 115 patients were reassessed. Association between NfL and clinical measures in PIGD and TD at baseline and at 2-year follow-up were analysed.

**Results:**

At baseline, plasma NfL levels were higher in PD than HC (8.8 ± 3.4 vs 16.2 ± 7.6 pg/ml, *p* < 0.0001), and differentiated PD from HC with a good diagnostic accuracy (AUC = 0.833, *p* < 0.001). At 2 years, NfL was higher in PIGD than TD (18.4 ± 14.5 vs 12.6 ± 4.4 pg/ml, *p* = 0.039). Within the PIGD group, higher NfL associated significantly with worse global cognition and UPDRS motor scores at baseline, and was able to predict motor and cognitive decline at a mean follow-up duration of 1.9 years, controlled for age, sex and disease duration.

**Conclusions:**

In this longitudinal study, we demonstrated for the first time the potential utility of plasma NfL as a diagnostic and prognostic biomarker in PIGD even at early stages of PD. These important novel findings will require further confirmation in larger, longitudinal PD cohorts.

## Background

Neuroaxonal loss remains the pathological substrate of permanent disability in neurological disorders. Reliable quantification of axonal loss is important for both disease monitoring and prognostication. The neurofilament proteins play an important role as their levels rise with neuroaxonal damage, indicating neuroaxonal injury independent of causal pathways [1]. Out of the neurofilament proteins, neurofilament light (NfL) chain has been recognized as a possible marker of axonal injury in various neurological disorders [1], including multiple sclerosis [2], motor neurone disease [3, 4], frontotemporal dementia spectrum disorders [5, 6], traumatic brain injury [7], and cerebrovascular disease [8]. In Parkinson’s disease (PD), blood NfL has been reported to be higher in cases vs controls [9, 10], and shown to be able to distinguish PD from atypical PD (APD) [11, 12]. NfL has also been found to associate with longer disease duration, more aggravated motor symptoms [11], and correlate with motor and/or cognitive decline [13] in PD.

Classification of PD motor subtypes based on the rate of clinical progression allows for both closer monitoring and earlier implementation of appropriate management for the poorer prognostic motor subtypes. The postural instability and gait disorder (PIGD) subtype has been associated with greater clinical progression and cognitive dysfunction, whereas tremor dominant (TD) PD is associated with a better prognosis [14, 15]. The heterogenous clinical phenotypes and varying disease course seen in PD patients highlights the need for biomarkers that can predict disease progression according to motor subtypes more accurately. Given the greater risk for cognitive impairment and motor progression in PIGD [14–16], we hypothesized that NfL may be a potential biomarker for the PIGD subtype. Furthermore, we aimed to determine if this association with PD motor subtypes can also be observed in early stages of the disease, which has important clinical implications for patient management and prognostication. To address these gaps, we conducted the first longitudinal study to investigate the association of NfL levels with cognitive and motor outcomes with PIGD and compared this with tremor-dominant PD.

## Methods

### Clinical recruitment

Participants were recruited from the National Neuroscience Institute, Singapore, between November 2014 and July 2018. All PD patients fulfilled the National Institute of Neurological Disorders and Stroke (NINDS) criteria for the diagnosis of PD. [17] The Early Parkinson’s Disease Longitudinal Singapore (PALS) study is an ongoing prospective cohort study analysing the progression of early PD over a follow-up period of 5 years. Patients were defined as ‘early PD’ using the following inclusion criteria: (i) motor symptoms ≤2 years, and (ii) carrying a diagnosis of PD of ≤1 year using the NINDS criteria as diagnosed by a movement disorders specialist. Healthy controls (HC) were recruited from the community and were free of significant neurological, psychiatric or systemic disease. Ethics approval was obtained from the Singapore Health Services Centralised Institutional Review Board (CIRB) for the use of human participants in this study, and all participants provided informed written consent.

### Clinical measures at baseline and follow-up

The Movement Disorders Society Unified Parkinson’s Disease Rating Scale (MDS-UPDRS) [18] was conducted by trained movement disorder specialists while participants were in the “ON” state, and scores were obtained thrice yearly. Functional status was determined using the Hoehn and Yahr (H&Y) rating scale [19] and motor severity using the MDS-UPDRS Part III motor components [18]. Patients were classified into motor subtypes of “tremor dominant” (TD), “postural instability and gait disorders” (PIGD) or “indeterminate” based on MDS-UPDRS part II and III components [20]. Patients classified as “indeterminate” were excluded from analysis. Global cognition was measured using the Mini-Mental State Examination (MMSE) [21] and Montreal Cognitive Assessment (MoCA) [22] tools.

### Plasma NfL measurement

Blood samples were collected at baseline and annually. A total of 199 participants (149 PD and 50 HC) had their plasma NfL levels measured at baseline. Seventy-six PD patients (30 TD and 46 PIGD) had plasma NfL measured at the 2-year mark. EDTA blood was centrifuged at 1500 g for 15 min within 1 h after collection. Plasma was aliquoted and stored at − 80 °C until further analysis. Samples were thawed only once for NfL measurement. NfL levels were measured using ultrasensitive single molecule array (Simoa) Human NfL assay and Simoa HD-1 Analyzer (Quanterix, MA), according to the manufacturer’s protocol. Coefficient of variation of samples measured in duplicates was < 10%. Two quality-control samples with high and low NfL concentrations, provided in the kit, were measured in each run and were in the expected range.

### Statistical analysis

Demographics and clinical characteristics were compared between PD and HC using independent two-sample t-test and Chi-square test for continuous and categorical variables, respectively. Correlations between clinical data and NfL levels were assessed using Spearman’s rank order correlation. The three motor subtypes (TD vs. PIGD vs. Indeterminate) were compared for similar parameters by analysis of variance (ANOVA) and Chi-square test for continuous and categorical variables, respectively. Natural logarithm (Ln) transformation was performed to reduce right skewness for plasma NfL levels for subsequent analysis. Multivariable linear regression analysis was performed to investigate the association between NfL levels (and its interaction with motor-subtype) and clinical outcomes, controlling for age, sex and disease duration as potential confounders. The diagnostic accuracy of plasma NfL (as a predictive marker of PD vs controls) was assessed via receiver operating characteristic (ROC) analysis. Multivariable logistic regression analysis was conducted to investigate the association between standardized Ln-NfL level (and its interaction with age) and clinical diagnosis (PD vs controls), and contour plot was generated based on the calculated predicted probabilities. Associations between baseline plasma NfL levels (and its interaction with motor subtypes) and change in motor and cognitive outcomes over time were investigated by linear mixed models, adjusted for age, sex and disease duration. Auto Regressive (AR-1) variance-covariance structure method was used to account for repeated measures outcomes. Statistical significance was set at *p* < 0.05, and all tests were two-sided. Statistical analysis was performed in SAS version 9.4 for Windows (SAS Institute Inc., Cary, NC, USA).

## Results

### Baseline characteristics

Participant demographics and clinical features at baseline are shown in Table 1. Plasma NfL positively correlated with age in PD (rs = 0.557, *p* < 0.001) and HC (rs = 0.294, *p* = 0.038), but not sex in both PD and HC. There was a negative association between NfL and disease duration (rs = − 0.291, *p* < 0.001) but not levodopa equivalent daily dosage (LEDD) (rs = 0.036, *p* = 0.676) in PD.

**Table 1:** Demographic and clinical characteristics of all subjects at baseline

Parameter	HC	PD	TD	PIGD	Indeterminate
*Subjects, n*	*50*	*149*	*70*	*55*	*24*
Age, years	57.2 ± 5.4^a^	64.0 ± 8.3^b**^	64.0 ± 8.0	63.6 ± 8.3	65.0 ± 9.4
Male sex, n(%)	25 (50)	85 (57)	43 (61)	28 (51)	14 (58)
Age at onset, years	–	62.9 ± 8.5	63.1 ± 8.2	62.3 ± 8.5	63.8 ± 9.4
Disease duration, years	–	1.1 ± 0.9	0.9 ± 0.7	1.3 ± 1.3^d*^	1.2 ± 0.6^c*^
LEDD	–	176.5 ± 135.3	172.6 ± 133.3	182.9 ± 142.4	172.3 ± 129.2
MDS-UPDRS Part II: ADLs	–	4.1 ± 3.2	3.7 ± 2.9	4.7 ± 3.5	4.0 ± 3.2
MDS-UPDRS Part III: Motor	–	21.4 ± 9.3	21.1 ± 10.0	22.0 ± 9.5	20.7 ± 7.0
H&Y stage,	–	1.8 ± 0.4	1.7 ± 0.4	1.9 ± 0.4^d*^	1.9 ± 0.4^c*^
MMSE	29.0 ± 1.3	26.8 ± 2.8^b**^	27.0 ± 2.5	26.6 ± 3.4	27.0 ± 2.0
MoCA	27.5 ± 2.0	25.1 ± 3.5^b**^	25.3 ± 3.3	24.5 ± 3.6	25.6 ± 4.2
Plasma NfL, pg/ml	8.8 ± 3.4	16.2 ± 7.6^b**^	15.8 ± 7.0	15.8 ± 7.1	18.2 ± 10.3

Abbreviations: *PD* Parkinson’s Disease; *HC* Healthy Control; *TD* Tremor-Dominant; *PIGD* Postural Instability Gait Disorder; *LEDD* Levodopa Equivalent Dose; *MDS-UPDRS* Movement Disorder Society Unified Parkinson’s Disease Rating Scale; *ADL* Activities of Daily Living; *H&Y stage* Hoehn and Yahr stage; *MMSE* Mini Mental State Examination; *MoCA* Montreal Cognitive Assessment; *NfL* Neurofilament light chain protein.^a^ Continuous variables reported as mean ± standard deviation; Categorical variables reported as n (%)

* *P* < 0.05, ** *P* < 0.01 using Analysis of variance for continuous variables; Chi-squared test for categorical variable for comparisons of variables between ^a^ PD and HC, ^c^ TD, PIGD and Indeterminate subtypes, ^d^ TD and PIGD subtypes

### Analyses between PD patients and HC

**Fig. 1:**
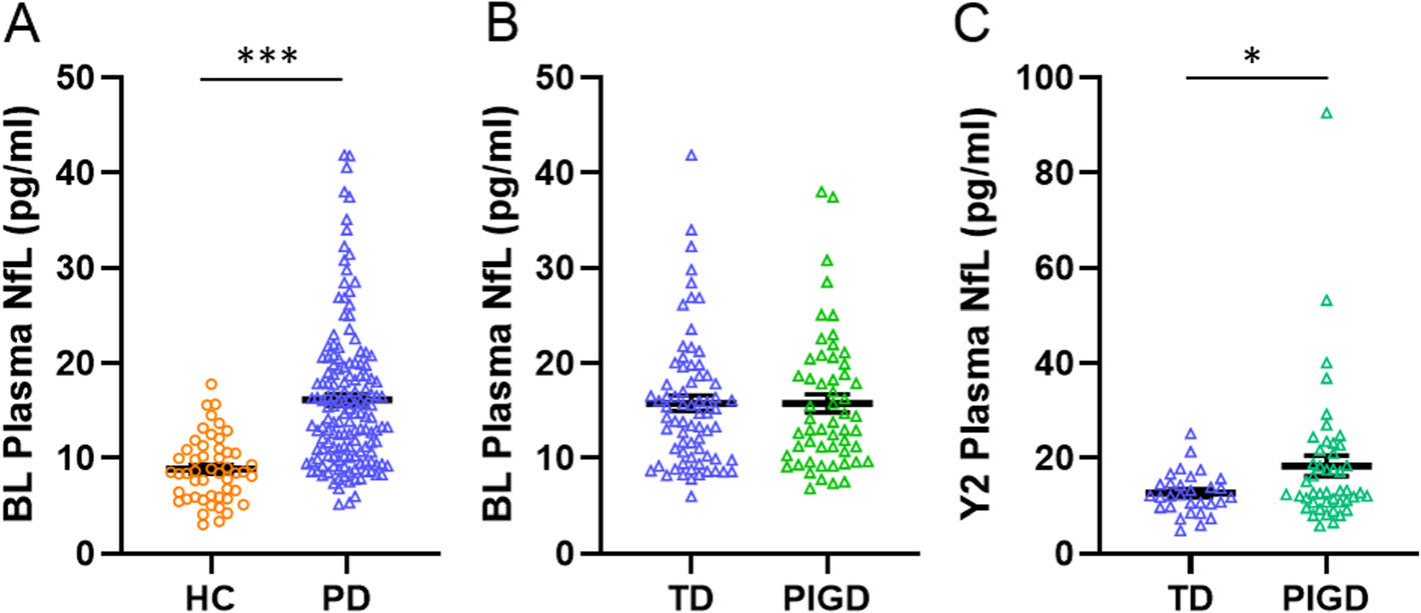
Plasma NfL in healthy controls and patients with Parkinson’s disease. **a** At baseline, plasma NfL was significantly increased in PD compared to HC, adjusting for age and sex; **b** but not between TD and PIGD, adjusting for age, sex and disease duration. **c** At year 2, plasma NfL was significantly increased in PIGD compared to TD, adjusting for age, sex and disease duration. Abbreviations: *BL,* Baseline; *PD*, Parkinson’s Disease; *HC*, Healthy Control; *NfL*, Neurofilament light chain protein; *TD*, Tremor-Dominant; *PIGD*, Postural Instability Gait Disorder; *Y2*, Year 2

**Fig. 2:**
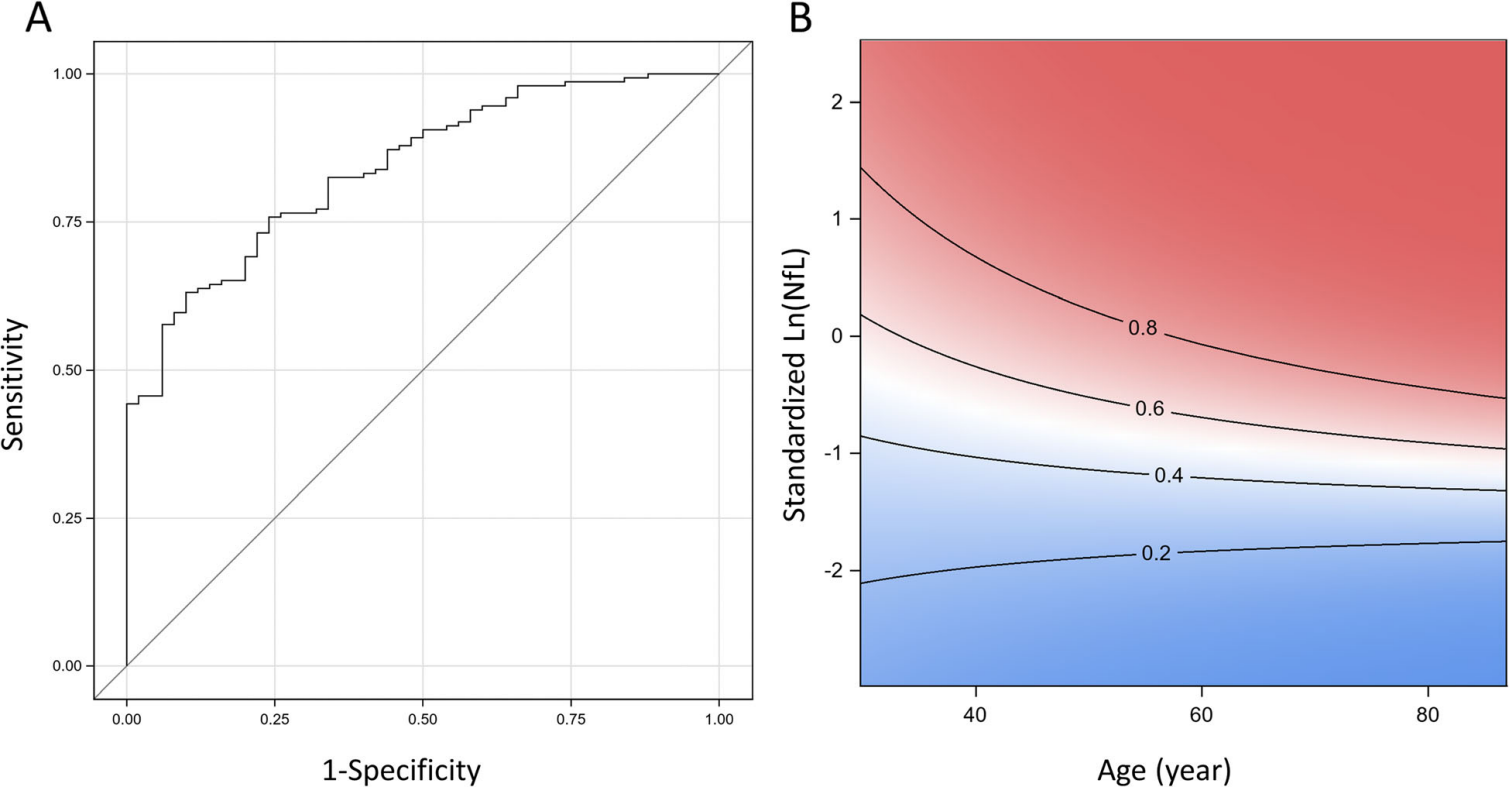
Baseline plasma NfL discriminates PD from healthy controls. **a** Receiver operating characteristic curve analysis in PD vs HC **b** Contour plot showing predicted probability of clinical diagnosis of PD at different NfL levels and age group. The darker shade of red indicates higher risk and darker blue indicates lower risk of PD. The solid line depicts the risk of PD using multivariable logistic regression model adjusted NfL x age interaction term. Abbreviations: *PD*, Parkinson’s Disease; *HC*, Healthy Control; *NfL*, Neurofilament light chain protein

Baseline plasma NfL levels were significantly elevated in PD compared to HC in univariate (*p* < 0.001; Table 1) and multivariable analyses (adjusted for age and sex, *p* < 0.001; Fig. 1a). ROC analysis showed that plasma NfL could discriminate PD from HC with area under the curve (AUC): 0.833, 95% CI: 0.774–0.893, sensitivity = 60%, specificity = 90%; Fig. 2).

### Analyses between PIGD and TD PD

While baseline plasma NfL levels did not differ significantly between TD and PIGD motor subtypes in univariate (Table 1) and multivariable analyses (adjusted for age, sex and disease duration, *p* = 0.687; Fig. 1b), we found that NfL levels were significantly higher in the PIGD than TD group at Year 2 (18.4 ± 14.5 vs 12.6 ± 4.4 pg/ml, *p* = 0.025, adjusted for age, sex and disease duration; Fig. 1cc). ROC analysis showed that plasma NfL could discriminate between the two motor subtypes with an AUC of 0.656, 95% CI: 0.533–0.779, sensitivity = 90%, specificity = 30.4%. Across all PD patients, NfL did not show significant association with cognitive or motor measures at baseline. In the PIGD group however, higher NfL levels significantly associated with worse MoCA and UPDRS Part III motor scores (Table 2), controlling for age, sex and disease duration. These results remained after adding LEDD as a covariate.

**Table 2:** Multivariable analysis on baseline plasma NfL levels with cognitive and motor outcomes in PD patients

Group	MDS-UPDRS Part III (Motor)^a^	H&Y	MoCA
	β^b^ (95% CI)	β (95% CI)	β (95% CI)
Total PD	−0.567 (−4.775, 3.642)	− 0.029 (− 0.214, 0.157)	−0.455 (− 1.995, 1.086)
TD	−6.815 (− 14.036, 0.407)	− 0.188 (− 0.498, 0.121)	1.437 (− 0.757, 3.631)
PIGD	10.687** (3.325, 18.050)	0.292 (− 0.021, 0.605)	−3.168* (− 5.954, − 0.382)

Abbreviations: *PD* Parkinson’s Disease; *TD* Tremor-Dominant; *PIGD* Postural Instability Gait Disorder; *H&Y stage* Hoehn and Yahr stage; *MDS-UPDRS* Movement Disorder Society Unified Parkinson’s Disease Rating Scale; *MoCA* Montreal Cognitive Assessment; *CI* Confidence Interval; *NfL*, Neurofilament light chain protein

^a^ MDS-UPDRS Part III (Motor) scores, H&Y stages and MoCA scores of PD patients are the outcome variables

^b^ Beta coefficient (and 95% CI) of Ln-NfL level; Multivariable linear regression adjusted for age, sex and disease duration

* *P* < 0.05, ** *P* < 0.01

### Two-year longitudinal outcomes between NfL levels and PD motor subtypes

Out of 149 PD patients, 115 patients (53 TD and 45 PIGD) with available follow-up data at 2 years were included in subsequent analysis to assess the prognostic utility of plasma NfL. The mean duration of follow-up was 1.9 years. Longitudinal data of plasma NfL, cognitive and motor scores are summarised in Table 3. Using linear mixed models, we tested the association between interaction of baseline plasma NfL levels with TD and PIGD subtypes and clinical outcomes in total PD over time, adjusting for baseline characteristics such as age, sex and disease duration. Across all PD patients, baseline plasma NfL did not associate significantly with motor and cognition outcome over time. However, higher baseline plasma NfL levels significantly associated with worse motor and cognition outcomes over time in the PIGD group (Table 4), but not in the TD group. In the PIGD group, every one unit increase in Ln-NfL (=2.72 increase in plasma NfL level) resulted in a corresponding 9.73 unit increase in motor outcome (as assessed by MDS-UPDRS part III) and 2.09 unit decrease in cognitive outcome (as assessed by MMSE), controlling for baseline age, sex and disease duration.

**Table 3:** Plasma NfL, cognitive and motor data in PD patients over different timepoints

Group	Values	Time			Trend *p* value
		Baseline	Year 1	Year 2	
Total PD	NfL	16.16 ± 0.62	–	16.11 ± 1.37	0.2881
	MMSE	26.82 ± 0.23	26.58 ± 0.25	26.9 ± 0.25	0.4523
	MoCA	25.06 ± 0.29	25.05 ± 0.31	25.17 ± 0.30	0.9324
	Motor	21.32 ± 0.76	24.76 ± 0.84	25.96 ± 1.03	< 0.001
TD	NfL	15.79 ± 0.83	–	12.62 ± 0.81	0.1865
	MMSE	26.99 ± 0.30	26.88 ± 0.28	27.13 ± 0.29	0.9128
	MoCA	25.32 ± 0.39	25.35 ± 0.39	25.42 ± 0.38	0.8401
	Motor	20.99 ± 1.19	24.40 ± 1.20	25.46 ± 1.37	< 0.001
PIGD	NfL	15.76 ± 0.96	–	18.39 ± 2.15	0.0474
	MMSE	26.55 ± 0.46	26.06 ± 0.54	26.5 ± 0.46	0.4865
	MoCA	24.47 ± 0.48	24.38 ± 0.63	24.61 ± 0.54	0.8705
	Motor	22.02 ± 1.28	26.17 ± 1.44	26.80 ± 1.77	< 0.001

Abbreviations: *PD* Parkinson’s Disease; *TD* Tremor-Dominant; *PIGD* Postural Instability Gait Disorder; *Motor* Movement Disorder Society Unified Parkinson’s Disease Rating Scale (MDS-UPDRS) part III scores; *MoCA* Montreal Cognitive Assessment; *NfL* Neurofilament light chain protein

Values represent the mean ± SD

**Table 4:** Multivariable analysis on baseline plasma NfL levels with cognitive and motor outcomes in PD patients over time

Outcomes	MDS-UPDRS Part III (Motor)^a^		MMSE	
	β (95% CI)^b^	*p* value	β (95% CI)	*p* value
NfL (PIGD)	9.73 (3.65, 15.81)	0.002	− 2.09 (−3.90, − 0.28)	0.024
NfL (TD)	−3.90 (− 9.36, 1.57)	0.161	0.50 (− 1.14, 2.13)	0.549
NfL x Motor Subtype	13.6 (6.16, 21.1)	< 0.001	−2.59 (− 4.81, − 0.36)	0.023
Age	0.10 (− 0.12, 0.32)	0.385	−0.08 (− 0.14, − 0.01)	0.025
Gender	−2.75 (− 5.84, 0.35)	0.081	−0.09 (− 1.01, 0.83)	0.839
Disease duration	−0.75 (− 2.43, 0.93)	0.379	0.17 (− 0.34, 0.69)	0.506

Abbreviations: *PD* Parkinson’s Disease; *TD* Tremor-Dominant; *PIGD* Postural Instability Gait Disorder; *MDS-UPDRS* Movement Disorder Society Unified Parkinson’s Disease Rating Scale; *MMSE* Mini Mental State Examination; *CI* Confidence Interval; *NfL* Neurofilament light chain protein

^a^ MDS-UPDRS Part III (Motor) and MMSE scores of PD patients over 2 years are the outcome variables

^b^ Beta coefficient (and 95% CI) of Ln-NfL level; Linear mixed models adjusted for age, sex, disease duration and NfL x motor subtype interaction

## Discussion

The PIGD motor subtype of PD is associated with a poorer prognosis and greater clinical progression in PD. In this 2-year longitudinal study, we investigated if plasma NfL may be a potential biomarker for the PIGD subtype of PD in early stages of disease. We demonstrated for the first time that higher NfL levels at baseline significantly associate with and predict greater global cognitive and motor impairment in patients with PIGD both at baseline and over 2 years, accounting for age, sex and disease duration. Furthermore, we were able to provide quantification of the extent to which baseline NfL can predict cognitive or motor decline within a relatively short follow-up period of 2 years.

While limited studies have previously reported higher blood NfL levels in PD compared to controls, our study has included a comparable number, if not substantially more participants than these [10–12]. Importantly, we were able to show that plasma NfL can discriminate PD from HC with high diagnostic accuracy even at early stages of the disease. NfL levels were measured in our patients at baseline, all of whom had a mean disease duration of 1.1 years at time of study recruitment, compared to other studies investigating NfL in PD which had mean disease duration ranging from 2 to 3 years [12], to 7 to 9 years [11, 13].

Additionally, NfL has previously shown only modest or inconsistent correlation with UPDRS Part III motor scores in PD [11, 13], possibly related to the fact that the PD groups in these studies were not analysed according to motor subtype. Higher NfL levels in PD can be explained given that NfL remains a well-established biomarker of neuroaxonal loss [1], and profound dopaminergic axonal loss has been shown to occur even at early stages of PD. [23] In vitro, induced pluripotent stem cell (iPSC)-derived dopaminergic neurons from patients carrying alpha-synuclein (*SNCA*) triplications or leucine-rich repeat kinase (*LRRK2*) G2019S mutations have also demonstrated evidence of neuroaxonal degeneration [24].

While plasma NfL levels were not different between TD and PIGD at baseline, this was not surprising given that both PD subtypes may appear similar at very early disease stages. In 2 years, a significant difference between NfL levels in both groups appeared, suggesting that PIGD may have progressed to a greater extent pathophysiologically than the more ‘benign’ TD subtype. Currently, there are no molecular biomarkers available that can reliably discriminate PD motor subtypes without reliance on clinical presentation, which often fluctuates and necessitate more objective biomarker-based discrimination. Multiple studies have investigated cerebrospinal fluid (CSF) biomarkers in PD motor subtypes; CSF Aβ-42 and P-tau181 concentrations have associated with the PIGD but not with TD or intermediate phenotypes [25], while CSF α-synuclein levels were found to be significantly lower in non-TD or PIGD phenotypes compared with the TD phenotype [26, 27]. Fewer studies, however, have attempted to identify blood-based biomarkers that can similarly discriminate between PD motor subtypes, a significant gap given the less invasive nature of blood sampling vs CSF sampling. Patients with the PIGD subtype were reported to have lower plasma Aβ-42 and higher plasma α -synuclein levels than the TD group [28], while our group has previously reported higher serum uric acid levels in the TD motor subtype [29]. Our results here suggest that approximately 3 years after symptom onset, plasma NfL may discriminate TD and PIGD subtypes, and potentially act as a diagnostic biomarker for PIGD, which will be useful particularly in the clinical trial setting.

Our findings are consistent with the known clinical hallmarks of PIGD, which is characterized by more severe disease manifestations at diagnosis, greater risk of cognitive impairment and neuropsychiatric symptoms than TD [14–16], and more rapid disease progression than the less malignant clinical subtype [30]. Importantly, clinical severity of PIGD has been proposed as a useful indicator of severity and prognosis in PD by itself [14], suggesting the more crucial need for a biomarker of disease intensity in PIGD, rather than in TD.

While we do not have postmortem confirmation of PD in the study, all our PIGD patients satisfied the clinical criteria for PD and were responsive to levodopa with no clinical features to suggest a Parkinson-Plus syndrome. At 2-year follow-up, clinical diagnosis of PIGD remained in this group of patients. Additionally, we accounted for LEDD in our analyses. Despite the moderate sensitivity of NfL in discriminating PD from HC, its high specificity remains clinically useful for initial screening of PD and PD subtypes from controls, together with other supportive clinical features with high sensitivity such as anosmia. Hence, NfL can be included in the initial workup of patients when diagnosis remains uncertain. The Early Parkinson’s Disease Longitudinal Singapore (PALS) study is an ongoing prospective cohort study analysing the progression of early PD over a follow-up period of 5 years. This longitudinal study will allow us to systematically analyse the performance of plasma NfL in combination with other biomarkers reflecting different pathophysiological processes in PD (e.g. plasma tau, alpha-synuclein and uric acid etc), which may improve its discriminatory performance. Additionally, blood samples collected at future timepoints will be analysed in the same sitting as far as possible to reduce batch-to-batch variability, with inter-assay control included in future runs.

## Conclusions

Here, we highlight a novel relationship between plasma NfL and PD motor subtypes by demonstrating an association between NfL and cognitive and motor measures over 2 years in PIGD. Overall, our results suggest that plasma NfL can be a potential biomarker to facilitate closer monitoring and management for PIGD, a poorer prognostic motor subtype of PD. The heterogeneity of PD clinical phenotypes with varying disease trajectories highlights the need for better biomarkers to facilitate more accurate monitoring and prognostication of PD subtypes, relevant in our push towards personalized medicine. These important findings offer great opportunities for further confirmation in larger, longitudinal PD cohorts.

## Data Availability

The datasets used and/or analysed in the current study are available from the corresponding author on reasonable request.

## Abbreviations

APD: Atypical PD
CSF: Cerebrospinal fluid
HC: Healthy controls
H&Y stage: Hoehn and Yahr stage
LEDD: Levodopa equivalent dose
MDS-UPDRS: Movement disorder society unified parkinson’s disease rating scale
MMSE: Mini mental state examination
MoCA: Montreal cognitive assessment
NINDS: National institute of neurological disorders and stroke
NfL: Neurofilament light chain protein
PALS: Parkinson’s disease longitudinal singapore
PD: Parkinson’s disease
PIGD: Postural instability gait disorder
ROC: Receiver operating characteristic
SIMOA: Single molecule array
SNCA: Alpha-synuclein
LRRK2: Leucine-rich repeat kinase
TD: Tremor-dominant
